# Efficacy of high-dose chemotherapy combined with hematopoietic stem cell transplantation in advanced neuroblastoma

**DOI:** 10.3389/fonc.2026.1819260

**Published:** 2026-05-14

**Authors:** Lin Wang, Jian Gao, Boshen Shu, Shufeng Zhang, Yinghao Sun, Xunqian Wang, Xiaohui Wang

**Affiliations:** Department of Pediatric Surgery, Henan Provincial People’s Hospital, Zhengzhou, Henan, China

**Keywords:** consolidation therapy, event-free survival, hematopoietic stem cell transplantation, high-dose chemotherapy, neuroblastoma, overall survival

## Abstract

**Background:**

High-dose chemotherapy plus hematopoietic stem cell transplantation (HDC+HSCT) is widely used as consolidation for advanced-stage neuroblastoma, but real-world comparative evidence versus high-dose chemotherapy (HDC) alone remains limited. This study aimed to compare the efficacy and safety of HDC+HSCT versus HDC alone in pediatric patients with advanced-stage neuroblastoma.

**Methods:**

In this retrospective cohort study, pediatric patients with advanced-stage neuroblastoma treated between October 2017 and October 2022 were identified from institutional medical records. Patients were classified according to the consolidation strategy actually delivered: HDC alone (n=108) or HDC+HSCT (n=103). The primary outcomes were overall survival (OS) and event-free survival (EFS), both calculated from the start of consolidation therapy. Secondary outcomes included objective response rate (ORR) at the first post-consolidation assessment and treatment-related toxicities. Survival was evaluated using Kaplan–Meier analysis and Cox proportional hazards models.

**Results:**

The HDC+HSCT group showed a higher ORR than the HDC group (78.6% vs 64.8%, P = 0.026). OS estimates were also higher in the HDC+HSCT group, with a median OS of 34.7 versus 26.1 months (log-rank P = 0.016); the adjusted hazard ratio (HR) for OS was 0.62 (95% confidence interval [CI] 0.41–0.94, P = 0.024). EFS did not differ significantly between groups (median, 21.9 vs 19.9 months; log-rank P = 0.937; adjusted HR 0.98, 95% CI 0.70–1.36, P = 0.901). Grade ≥3 bacterial infection (35.0% vs 22.2%, P = 0.040), sepsis (11.7% vs 1.9%, P = 0.004), mucositis (31.1% vs 17.6%, P = 0.022), and renal toxicity (9.7% vs 1.9%, P = 0.014) were more frequent in the HDC+HSCT group.

**Conclusion:**

HDC+HSCT was associated with higher post-consolidation ORR and more favorable OS estimates than HDC alone, while EFS was similar between groups. It was also associated with more selected severe toxicities, underscoring the need for intensive supportive care. Prospective studies are needed to validate these findings.

## 1 Introduction

Neuroblastoma remains the most common extracranial solid malignancy of early childhood and accounts for a disproportionate share of pediatric cancer mortality (1). Advanced-stage disease, particularly metastatic presentations that meet contemporary high-risk criteria, continues to pose a therapeutic challenge despite iterative improvements in multimodality care (2). Recent overviews emphasize that risk stratification is increasingly refined by integrating clinical staging, tumor biology, and treatment-era effects, with ongoing international efforts aimed at optimizing risk classification to better align intensity with relapse risk (3, 4). The Children’s Oncology Group research agenda has highlighted persistent relapse as the dominant cause of treatment failure in high-risk neuroblastoma and prioritizes strategies that deepen remission and improve durable disease control across the consolidation and maintenance phases (5, 6).

Consolidation therapy is intended to eradicate residual disease that persists after induction, surgery, and local control. High-dose chemotherapy (HDC) with hematopoietic stem cell rescue has been used to enable myeloablative or near-myeloablative intensification, reflecting the chemosensitivity of neuroblastoma and the dose–response relationship of several cytotoxic agents (7). Nevertheless, the benefit of transplant-intensified consolidation in the contemporary era remains clinically relevant to re-evaluate because high-risk treatment sequences have evolved substantially, particularly with broader incorporation of immunotherapy and response-adaptive approaches (8). A recent systematic literature review comparing myeloablative therapy with autologous stem cell transplantation versus non-transplant strategies, including analyses in the context of subsequent anti-disialoganglioside GD2 (anti-GD2) therapy, underscores that consolidation choices may influence survival but that real-world variability in sequencing and supportive care complicates direct inference (9). Consistent with this, recent pediatric oncology reports have explored response-adapted consolidation strategies based on early metastatic response, supporting the concept that the consolidation platform may interact with disease burden and chemosensitivity at the end of induction (9, 10).

A central biological and clinical problem in high-risk neuroblastoma is minimal residual disease, which may not be apparent on routine imaging yet can seed subsequent relapse (11). Contemporary MRD-focused work highlights both the prevalence of residual disease after initial remission and its association with later events, motivating intensified consolidation for selected patients and more sensitive post-treatment monitoring (12). Current clinical guidance also recognizes that outcomes after relapse remain limited overall, reinforcing the importance of consolidation strategies that reduce early progression and enable effective downstream therapy. At the same time, intensification must be balanced against regimen-related toxicity and late effects, which remain substantial in survivors treated with transplant-based approaches (13, 14). Given these considerations, we conducted a single-center retrospective cohort study to evaluate the efficacy and safety of HDC combined with HSCT compared with HDC alone in pediatric patients with advanced-stage neuroblastoma treated with consolidation-intent therapy, focusing on post-consolidation response, survival endpoints, and clinically relevant toxicities.

## 2 Methods

### 2.1 Study design

This retrospective cohort study enrolled pediatric patients diagnosed with advanced-stage neuroblastoma who were treated between October 2017 and October 2022. Eligible patients were identified from institutional medical records and assigned to groups according to the consolidation strategy received during the study period: patients who underwent HDC alone were classified as the control group, whereas those who received HDC combined with hematopoietic stem cell transplantation (HSCT) were classified as the transplantation group. Inclusion criteria were: (1) age <18 years at diagnosis; (2) pathologically confirmed neuroblastoma with advanced-stage disease (International Neuroblastoma Staging System stage 3 with unfavorable biology or stage 4, as documented in the medical record); (3) receipt of protocol-based induction therapy followed by HDC with or without HSCT at our institution; and (4) availability of complete baseline clinicopathological data and follow-up information sufficient to ascertain study endpoints. Exclusion criteria were: (1) prior malignancy or concomitant malignant disease; (2) receipt of HSCT before the index HDC course; (3) severe uncontrolled organ dysfunction precluding HDC and/or HSCT (including but not limited to refractory heart failure, decompensated hepatic failure, or end-stage renal disease) documented before treatment allocation; (4) active uncontrolled infection at the time of HDC/HSCT; and (5) incomplete key variables or loss to follow-up before outcome assessment. The study was approved by the Ethics Committee of our hospital and conducted in accordance with the Declaration of Helsinki. Informed consent was obtained from legal guardians. All data were anonymized prior to analysis.

### 2.2 Grouping and treatment protocol

Patients were grouped according to the consolidation strategy actually delivered, as confirmed from chemotherapy prescriptions and transplant-unit documentation. The control group received HDC alone during consolidation, without subsequent stem cell infusion. The HDC+HSCT group received myeloablative HDC followed by HSCT within the same consolidation phase. The grouping strategy was based on the consolidation treatment actually delivered in our institution. This approach is consistent with the international literature, in which carboplatin, etoposide, and melphalan (CEM) and busulfan and melphalan (BuMel) are established high-dose regimens and transplant-based consolidation is a recognized strategy for high-risk neuroblastoma (15–18). In both groups, the high-dose consolidation chemotherapy regimens comprised either CEM or BuMel. For a given regimen, the planned drug components, target doses, and dosing schedules were identical between groups; the key between-group difference was whether HSCT was subsequently performed. These supportive-care procedures were delivered according to unified institutional protocols by the same pediatric oncology, hematology-transplant, and related supportive-care teams throughout the study period. Accordingly, apart from transplantation-related procedures inherent to HSCT, including stem cell collection, stem cell infusion, engraftment monitoring, and transplantation-related inpatient management, no other protocol-defined between-group differences were present during the consolidation phase. For CEM, carboplatin and etoposide were administered over four consecutive days, and melphalan was administered over three consecutive days immediately thereafter (total doses: carboplatin 1500 mg/m², etoposide 1200 mg/m², and melphalan 180 mg/m²). For BuMel, busulfan was administered on days −6 to −3 (intravenous dosing with pharmacokinetic-guided adjustment), followed by melphalan on days −2 to −1 prior to stem cell infusion. Supportive care was standardized and recorded, including pre-conditioning organ-function assessment and infection screening, antimicrobial prophylaxis, transfusion support, growth factor administration, mucositis and antiemetic management, fluid and electrolyte monitoring, and nutritional support; anticonvulsant prophylaxis was used for busulfan-based conditioning per institutional practice.

Overall treatment in this cohort generally consisted of protocol-based induction chemotherapy, followed by local control measures as clinically indicated, including surgery and/or radiotherapy, and then consolidation treatment with HDC with or without HSCT according to the strategy actually delivered in routine clinical practice. During the treatment course, anti-GD2 immunotherapy was not routinely available at our center and was therefore not administered as part of maintenance treatment in this cohort. Post-consolidation management did not differ between the two groups and mainly consisted of follow-up surveillance, with retinoid-based differentiation therapy and/or radiotherapy administered in selected patients according to institutional practice.

### 2.3 Data collection

Data were retrospectively collected using a standardized case report form from the electronic medical record, chemotherapy prescribing system, operative and pathology reports, radiotherapy records, transplant-unit documentation, laboratory information system, imaging archive, and institutional follow-up database. Baseline demographic and clinical variables included age at diagnosis, sex, body weight and body surface area, and presenting symptoms and physical findings documented at initial evaluation. Tumor-related characteristics included stage determined according to the International Neuroblastoma Staging System (INSS), primary tumor site, and metastatic sites (including bone, bone marrow, liver, lymph nodes, and other organs) as documented by staging work-up and imaging. Biological variables comprised MYCN amplification status, 1p and 11q chromosomal aberrations, and DNA ploidy. Baseline serum biomarkers were recorded from pretreatment testing, including lactate dehydrogenase, neuron-specific enolase, and ferritin.

Treatment-related variables included the induction chemotherapy regimen and number of induction cycles, extent of surgical resection classified as gross total resection, near-total resection, subtotal resection, or biopsy only, and whether radiotherapy, immunotherapy, and retinoid-based differentiation therapy were administered, with dates recorded. Consolidation treatment variables captured HDC details, including regimen components, planned and delivered doses, cycle dates and intervals, and treatment delay or dose reduction with the documented indication. For patients undergoing HSCT, transplant variables included conditioning regimen, stem cell source, infused CD34+ cell dose, time to neutrophil and platelet engraftment, duration of severe neutropenia, and transplantation-related length of hospital stay. To further characterize recovery after consolidation, hematologic recovery metrics were additionally collected for both groups, including time to neutrophil recovery, time to platelet recovery, duration of grade 4 neutropenia, and the timing of initiation of planned post-consolidation therapy, with reasons for delay recorded when applicable.

Outcome-related variables were extracted in accordance with prespecified endpoint definitions, including response assessment results, date of relapse or progression, survival status, and treatment-related toxicities and complications. Follow-up was conducted through scheduled outpatient visits, telephone interviews, readmission review, and electronic chart verification. Patients were followed every 3 months during the first 2 years after completion of treatment and every 6 months thereafter. Follow-up ended in October 2025 or at the last confirmed contact. Patients without any documented contact beyond the scheduled follow-up interval were considered lost to follow-up and were censored at the date of last confirmed survival. Missing data were handled using complete-case analysis; multiple imputation was performed as a sensitivity analysis for key covariates.

### 2.4 Outcome measures and assessment

The primary outcomes were overall survival (OS) and event-free survival (EFS). OS was defined as the interval from the start of consolidation therapy (date of first HDC administration) to death from any cause; patients alive at the last follow-up were censored on the date of last confirmed contact. EFS was defined as the interval from the start of consolidation therapy to the first occurrence of any predefined event, including disease relapse, disease progression, second malignant neoplasm, or death from any cause, whichever occurred first; patients without an event were censored at the last follow-up date. Disease status was assessed according to institutional neuroblastoma response evaluation procedures based on serial clinical assessments, imaging studies (contrast-enhanced CT or MRI for primary and soft-tissue disease), metastatic evaluation (including bone marrow examinations and functional imaging when performed as part of routine care), and tumor marker trends (including neuron-specific enolase) at prespecified post-treatment time points and during surveillance.

Secondary outcomes included objective response rate (ORR), defined as the proportion of patients achieving complete response (CR) or partial response (PR) at the first formal response assessment after completion of consolidation therapy, and depth of response, summarized as the distribution of response categories (CR, PR, stable disease, and progressive disease). Treatment-related toxicities were captured from hospitalization and follow-up records and graded according to the Common Terminology Criteria for Adverse Events (CTCAE). Toxicity domains of interest included hematologic toxicity (neutropenia, thrombocytopenia, anemia, and febrile neutropenia), documented infections, gastrointestinal and mucosal toxicity (including mucositis), hepatic complications including veno-occlusive disease/sinusoidal obstruction syndrome (VOD/SOS), and renal toxicity; for each event, the highest CTCAE grade within the consolidation period was recorded. Neutrophil recovery was defined as the first of 3 consecutive days with an absolute neutrophil count ≥0.5 × 10^9/L. Platelet recovery was defined as the first day with a platelet count ≥20 × 10^9/L without transfusion support for 7 days. Duration of grade 4 neutropenia was defined as the number of days during which the neutrophil count remained within the CTCAE grade 4 range during the consolidation period. Time to initiation of planned post-consolidation therapy was defined as the interval from completion of consolidation to the start of planned post-consolidation therapy. Delayed initiation of planned post-consolidation therapy was defined as failure to start planned post-consolidation therapy within the institutionally scheduled interval after completion of consolidation.

### 2.5 Statistical Analysis

All statistical analyses were performed using IBM SPSS Statistics, version 28.0 (IBM Corp., Armonk, NY, USA). Continuous variables are presented as mean ± standard deviation or median (interquartile range), as appropriate, and were compared using the independent-samples t test or Mann–Whitney U test. Categorical variables are summarized as counts and percentages and were compared using the χ² test; Fisher’s exact test was applied when expected cell counts were small. Time-to-event outcomes were analyzed using the Kaplan–Meier method, with between-group differences evaluated by the log-rank test. OS and EFS were further examined using Cox proportional hazards regression, and results are reported as hazard ratios (HRs) with 95% confidence intervals (CIs). Treatment response outcomes, including ORR, and binary safety endpoints, including prespecified grade ≥3 adverse events, were analyzed using logistic regression, with results reported as odds ratios (ORs) with 95% CIs. Subgroup analyses were conducted by fitting Cox models within strata and evaluating treatment-by-subgroup interaction terms.

## 3 Results

### 3.1 Patient selection and study flow

A total of 237 pediatric patients with advanced neuroblastoma were screened for eligibility. After verification of clinical records, chemotherapy prescriptions, and transplant-unit documentation, 26 patients were excluded according to prespecified criteria, and 211 patients were included in the final analysis. The final cohort comprised 108 patients in the HDC group and 103 patients in the HDC+HSCT group. Reasons for exclusion were as follows: (1) consolidation therapy was not completed as defined by the protocol or the delivered consolidation strategy could not be classified as HDC or HDC+HSCT (n=10); (2) key baseline or exposure data required for variable definition were missing (n=9), including incomplete documentation of INSS stage, essential biological markers, or consolidation treatment dates/dose information; and (3) outcome ascertainment was not feasible due to insufficient follow-up information (n=7), including absence of a formal response assessment or inability to verify relapse/progression timing or survival status.

### 3.2 Baseline characteristics and tumor profile

Baseline demographic and clinical characteristics were generally comparable between the HDC group (n=108) and the HDC+HSCT group (n=103). There were no significant between-group differences in age at diagnosis, body weight, or body surface area (all P>0.05). The proportion of male patients was lower in the HDC+HSCT group than in the HDC group (46.6% vs 64.8%; χ²=7.09, P = 0.008). With respect to presenting features, abdominal mass, fever, anemia-related manifestations, and neurologic symptoms occurred at similar frequencies (all P>0.05), whereas bone pain/limp was more frequently documented in the HDC+HSCT group (44.7% vs 31.5%; χ²=3.89, P = 0.049). Tumor characteristics were balanced between groups, including INSS stage distribution (χ²=0.83, P = 0.362), primary tumor site distribution (χ²=1.94, P = 0.584), and metastatic involvement across evaluated sites (all P>0.05). Tumor biological variables (MYCN amplification, 1p and 11q aberrations, and DNA ploidy) did not differ significantly between groups (all P>0.05). Baseline serum biomarkers measured before treatment, including lactate dehydrogenase, neuron-specific enolase, and ferritin, were also comparable between groups (all P>0.05) (**Table 1**).

**Table 1:** Baseline clinical characteristics and tumor profile.

Domain	Variable	HDC (n=108)	HDC+HSCT (n=103)	Test statistic	P value
Demographics	Age at diagnosis, years	4.88 ± 2.29	4.47 ± 2.51	t=1.23	0.221
	Weight, kg	18.36 ± 6.11	17.17 ± 5.41	t=1.50	0.135
	Body surface area, m²	0.76 ± 0.20	0.74 ± 0.20	t=0.95	0.343
	Male sex, n (%)	70 (64.8)	48 (46.6)	χ²=7.09	0.008
Presenting features	Abdominal mass, n (%)	75 (69.4)	74 (71.8)	χ²=0.15	0.702
	Bone pain/limp, n (%)	34 (31.5)	46 (44.7)	χ²=3.89	0.049
	Fever, n (%)	33 (30.6)	23 (22.3)	χ²=1.83	0.176
	Anemia-related manifestations, n (%)	37 (34.3)	29 (28.2)	χ²=0.91	0.339
	Neurologic symptoms, n (%)	13 (12.0)	16 (15.5)	χ²=0.54	0.461
Tumor stage	INSS stage III, n (%)	30 (27.8)	23 (22.3)		
	INSS stage IV, n (%)	78 (72.2)	80 (77.7)		
	INSS stage (overall)			χ²=0.83	0.362
Primary site	Adrenal, n (%)	71 (65.7)	62 (60.2)		
	Retroperitoneal (non-adrenal), n (%)	14 (13.0)	17 (16.5)		
	Mediastinal, n (%)	15 (13.9)	19 (18.4)		
	Other, n (%)	8 (7.4)	5 (4.9)		
	Primary site (overall)			χ²=1.94	0.584
Metastatic sites	Bone, n (%)	59 (54.6)	65 (63.1)	χ²=1.56	0.211
	Bone marrow, n (%)	51 (47.2)	52 (50.5)	χ²=0.22	0.635
	Liver, n (%)	23 (21.3)	23 (22.3)	χ²=0.03	0.856
	Lymph nodes, n (%)	34 (31.5)	36 (35.0)	χ²=0.29	0.593
	Other organs, n (%)	19 (17.6)	13 (12.6)	χ²=1.01	0.314
Tumor biology	MYCN amplification, n (%)	34 (31.5)	40 (38.8)	χ²=1.25	0.263
	1p aberration, n (%)	33 (30.6)	23 (22.3)	χ²=1.83	0.176
	11q aberration, n (%)	34 (31.5)	30 (29.1)	χ²=0.14	0.710
	Hyperdiploid DNA ploidy, n (%)	46 (42.6)	39 (37.9)	χ²=0.49	0.484
Baseline serum biomarkers	Lactate dehydrogenase, U/L	842.62 ± 245.91	909.13 ± 277.20	t=-1.84	0.067
	Neuron-specific enolase, ng/mL	128.63 ± 47.92	138.32 ± 58.46	t=-1.31	0.191
	Ferritin, ng/mL	468.40 ± 214.75	482.95 ± 221.18	t=-0.50	0.617

HDC, high-dose chemotherapy; HSCT, hematopoietic stem cell transplantation; BSA, body surface area; INSS, International Neuroblastoma Staging System.

### 3.3 Treatment response at the first post-consolidation assessment

At the first formal evaluation after consolidation, the ORR was higher in the HDC+HSCT group than in the HDC group (78.6% vs 64.8%; χ²=4.95, P = 0.026). Complete response was observed more frequently in the HDC+HSCT group than in the HDC group (38.8% vs 25.9%), whereas progressive disease was less frequent (6.8% vs 13.0%). When response depth was analyzed across all four categories (CR, PR, SD, and PD), the overall distribution did not differ significantly between groups (χ²=6.43, P = 0.093), although the observed pattern was consistent with deeper responses and fewer progressions in the HDC+HSCT group (**Table 2**).

**Table 2:** Objective response and depth of response at the first post-consolidation assessment.

Outcome	HDC (n=108)	HDC+HSCT (n=103)	Test statistic	P value
Time to first response assessment, weeks	4.31 ± 1.11	4.10 ± 0.97	t=1.50	0.135
CR, n (%)	28 (25.9)	40 (38.8)		
PR, n (%)	42 (38.9)	41 (39.8)		
SD, n (%)	24 (22.2)	15 (14.6)		
PD, n (%)	14 (13.0)	7 (6.8)		
Response category distribution (CR/PR/SD/PD), overall			χ²=6.43	0.093
ORR (CR+PR), n (%)	70 (64.8)	81 (78.6)	χ²=4.95	0.026

HDC, high-dose chemotherapy; HSCT, hematopoietic stem cell transplantation; ORR, objective response rate; CR, complete response; PR, partial response; SD, stable disease; PD, progressive disease.

### 3.4 Overall survival and event-free survival

**Figure 1:**
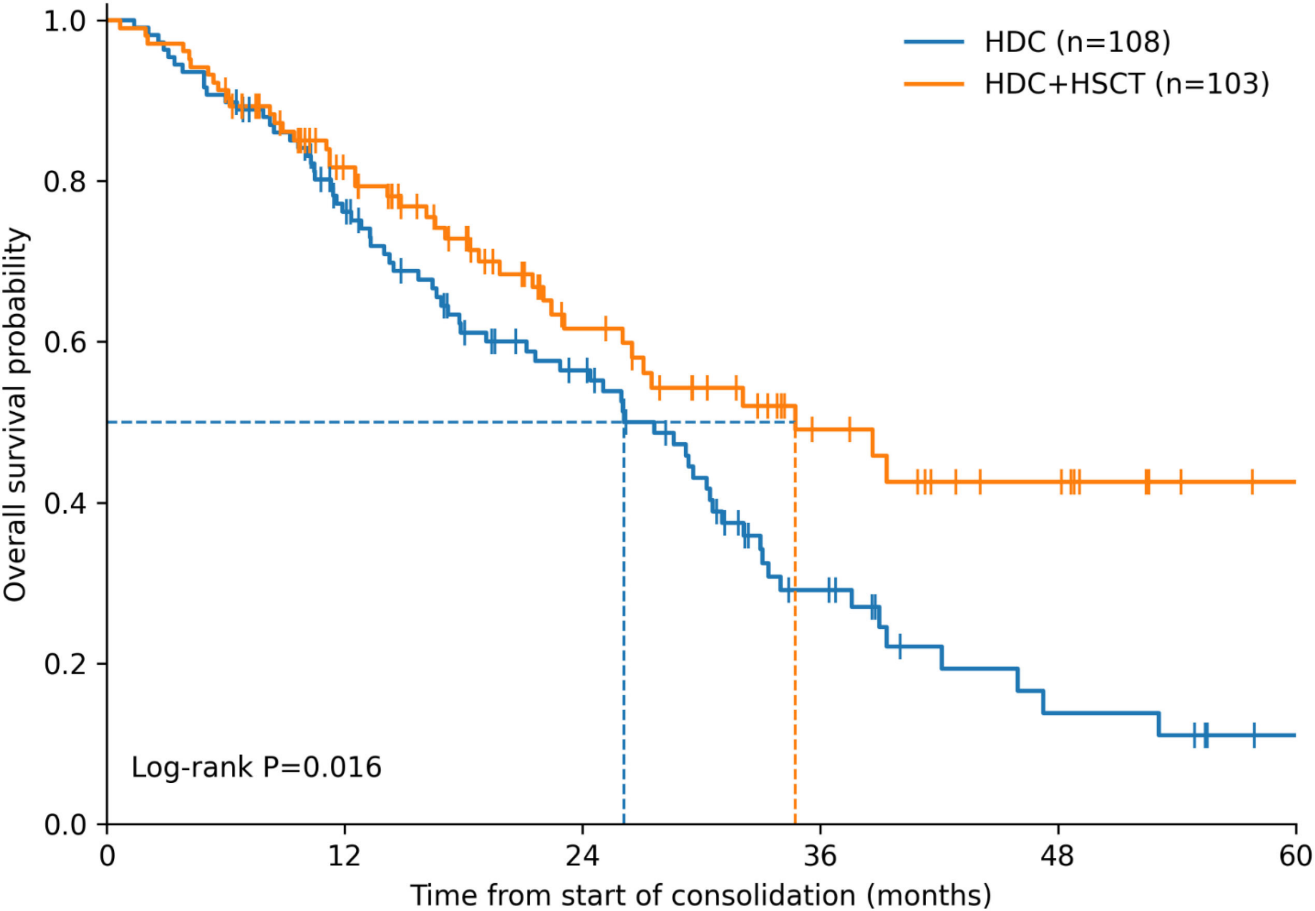
Overall survival by consolidation strategy. Kaplan–Meier estimates of overall survival (OS) from the start of consolidation therapy are shown for the high-dose chemotherapy (HDC) group (n=108) and the high-dose chemotherapy plus hematopoietic stem cell transplantation (HDC+HSCT) group (n=103). OS was higher in the HDC+HSCT group than in the HDC group (log-rank χ²=5.84, P = 0.016). The estimated 1-, 3-, and 5-year OS rates were 81.7%, 49.1%, and 42.6% for HDC+HSCT versus 76.2%, 29.1%, and 11.0% for HDC; median OS was 34.7 months and 26.1 months, respectively. Tick marks indicate censored observations.

**Figure 2:**
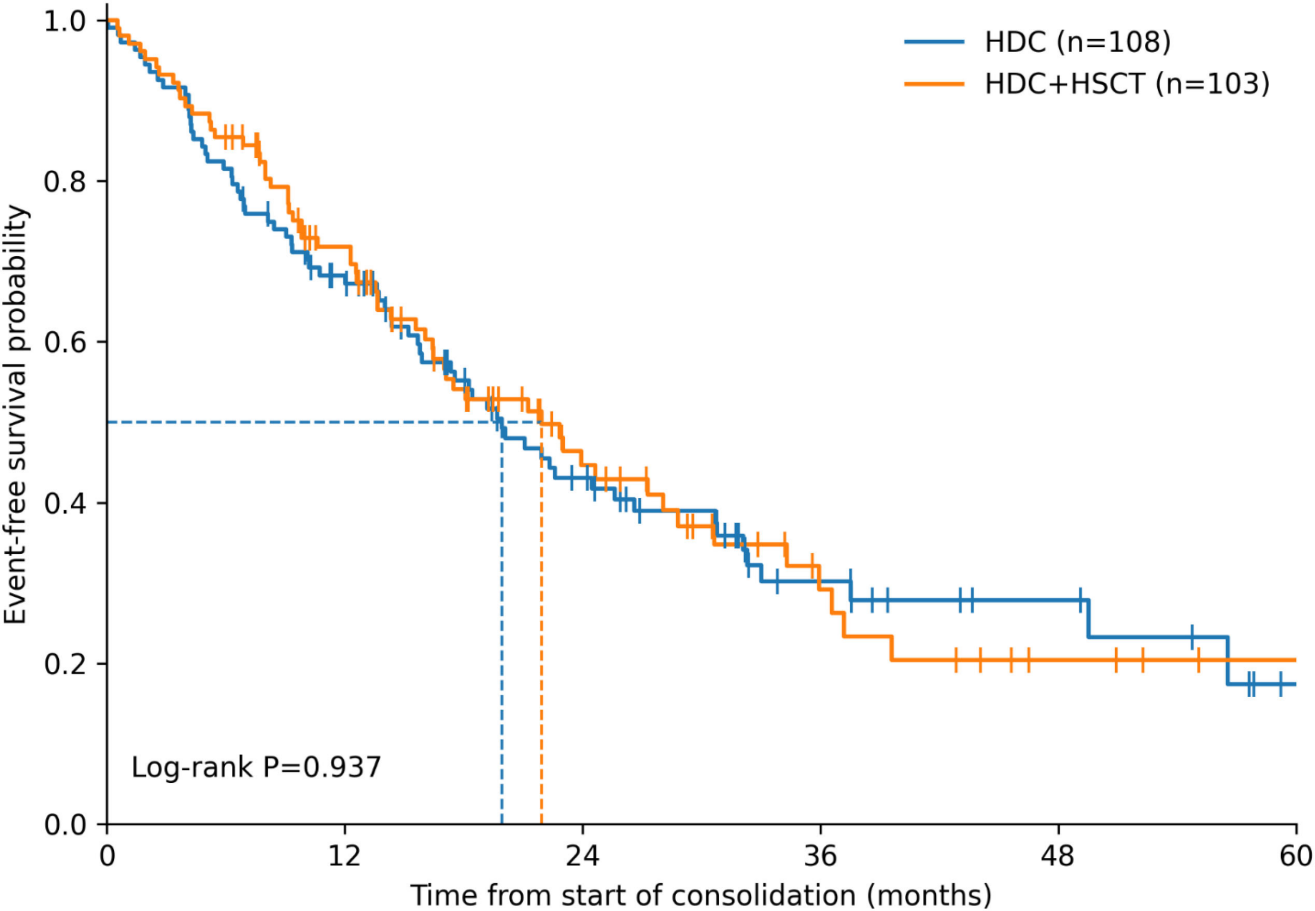
Event-free survival by consolidation strategy. Kaplan–Meier estimates of event-free survival (EFS) from the start of consolidation therapy are shown for the HDC group (n=108) and the HDC+HSCT group (n=103). EFS did not differ significantly between groups (log-rank χ²=0.01, P = 0.937). Median EFS was 21.9 months in the HDC+HSCT group and 19.9 months in the HDC group. Events contributing to EFS included relapse, progression, second malignant neoplasm, and death; relapse comprised the largest proportion in both groups. Tick marks indicate censored observations.

Kaplan–Meier curves for OS and EFS are presented in **Figure 1** and **Figure 2**, respectively. The HDC+HSCT group had higher OS estimates than the HDC group (log-rank χ²=5.84, P = 0.016). The estimated 1-, 3-, and 5-year OS rates were 81.7%, 49.1%, and 42.6% in the HDC+HSCT group, compared with 76.2%, 29.1%, and 11.0% in the HDC group; median OS was 34.7 months and 26.1 months, respectively. In contrast, EFS did not differ between groups (log-rank χ²=0.01, P = 0.937). Median EFS was 21.9 months in the HDC+HSCT group and 19.9 months in the HDC group, with similar 1-, 3-, and 5-year EFS estimates (**Table 3**). Among EFS events, relapse accounted for the largest proportion in both groups, followed by progression, with second malignant neoplasms and deaths comprising smaller fractions (**Table 4**).

**Table 3:** Kaplan–Meier estimates for OS and EFS.

Endpoint	Time point (months)	HDC (n=108)	HDC+HSCT (n=103)
OS	12	76.2 (66.8–83.2)	81.7 (72.4–88.1)
OS	36	29.1 (19.2–39.7)	49.1 (36.0–61.0)
OS	60	11.0 (4.0–22.2)	42.6 (28.6–55.8)
OS	Median (months)	26.1	34.7
OS	Log-rank	χ²=5.84	P=0.016
EFS	12	68.3 (58.5–76.2)	71.8 (61.8–79.6)
EFS	36	30.2 (20.2–40.8)	29.2 (17.9–41.4)
EFS	60	17.4 (6.6–32.5)	20.4 (10.2–33.1)
EFS	Median (months)	19.9	21.9
EFS	Log-rank	χ²=0.01	P=0.937

OS, overall survival; EFS, event-free survival; HDC, high-dose chemotherapy; HSCT, hematopoietic stem cell transplantation.

**Table 4:** EFS event decomposition.

Event type	HDC, n (%)	HDC+HSCT, n (%)
Relapse	38 (56.7)	28 (47.5)
Progression	15 (22.4)	18 (30.5)
Second malignant neoplasm	5 (7.5)	1 (1.7)
Death	9 (13.4)	12 (20.3)
Total EFS events	67	59

EFS, event-free survival; HDC, high-dose chemotherapy; HSCT, hematopoietic stem cell transplantation.

### 3.5 Safety outcomes

During consolidation, grade 3–4 neutropenia was frequent in both groups (91.7% in the HDC group vs 96.1% in the HDC+HSCT group; P = 0.179), and rates of grade 3–4 thrombocytopenia and anemia were numerically higher in the HDC+HSCT group but did not reach statistical significance (P = 0.079 and P = 0.204, respectively). Infections were common, and the rate of documented bacterial infection was higher in the HDC+HSCT group than in the HDC group (35.0% vs 22.2%; P = 0.040). Sepsis was also observed more frequently in the HDC+HSCT group (11.7% vs 1.9%; P = 0.004). Grade ≥3 mucositis was more frequent in the HDC+HSCT group (31.1% vs 17.6%; P = 0.022). Hepatic VOD/SOS was observed in 9.7% of patients in the HDC+HSCT group and 3.7% in the HDC group (P = 0.080). Grade ≥3 renal toxicity was uncommon overall but was observed more frequently in the HDC+HSCT group (9.7% vs 1.9%; P = 0.014). ICU admission was rare and did not differ significantly between groups (P = 0.370). The median time to neutrophil recovery was longer in the HDC+HSCT group than in the HDC group (15 (10–15) vs 13 (10–13, 19) days, P = 0.018), and the median time to platelet recovery was likewise prolonged (22 (14–18, 20–26) vs 18 (11–18, 20–22) days, P = 0.011). The duration of grade 4 neutropenia was also longer in the HDC+HSCT group (9 (6–9, 19) vs 7 (4–9) days, P = 0.020). Among patients scheduled to receive post-consolidation radiotherapy and/or retinoid-based maintenance therapy, the median interval from completion of consolidation to initiation of planned post-consolidation therapy was 52 (45–61) days in the HDC+HSCT group and 46 (39–55) days in the HDC group (P = 0.067). Delayed initiation of planned post-consolidation therapy occurred in 27.0% of patients in the HDC+HSCT group and 19.4% in the HDC group (P = 0.279) (**Table 5**).

**Table 5:** Treatment-related toxicities and complications during consolidation.

Adverse event (highest grade during consolidation)	HDC (n=108), n (%)	HDC+HSCT (n=103), n (%)	Test statistic	P value
Grade 3–4 neutropenia	99 (91.7)	99 (96.1)	χ²=1.81	0.179
Grade 3–4 thrombocytopenia	71 (65.7)	79 (76.7)	χ²=3.08	0.079
Grade 3–4 anemia	41 (38.0)	48 (46.6)	χ²=1.61	0.204
Febrile neutropenia	36 (33.3)	42 (40.8)	χ²=1.25	0.263
Any documented infection (bacterial/fungal/viral)	36 (33.3)	34 (33.0)	χ²=0.00	0.960
Bacterial infection	24 (22.2)	36 (35.0)	χ²=4.20	0.040
Fungal infection	10 (9.3)	11 (10.7)	χ²=0.12	0.730
Viral infection	10 (9.3)	14 (13.6)	χ²=0.98	0.322
Sepsis	2 (1.9)	12 (11.7)	χ²=8.17	0.004
ICU admission	4 (3.7)	1 (1.0)	Fisher exact	0.370
Grade ≥3 mucositis	19 (17.6)	32 (31.1)	χ²=5.22	0.022
VOD/SOS (any grade)	4 (3.7)	10 (9.7)	χ²=3.07	0.080
Grade ≥3 renal toxicity (AKI/creatinine increase)	2 (1.9)	10 (9.7)	χ²=6.07	0.014
Hematologic recovery after consolidation, median (IQR)				
Time to neutrophil recovery, days	13 (11–16)	15 (13–18)	U=4562	0.018
Time to platelet recovery, days	18 (14–24)	22 (17–28)	U=4387	0.011
Duration of grade 4 neutropenia, days	7 (5–10)	9 (7–12)	U=4475	0.020
Initiation of planned post-consolidation therapy				
Patients scheduled to receive radiotherapy and/or retinoid therapy, n (%)	72 (66.7)	74 (71.8)	χ²=0.65	0.420
Time to initiation of next planned treatment, days	46 (39–55)	52 (45–61)	U=2231	0.067
Delayed initiation of planned treatment, n/N (%)	14/72 (19.4)	20/74 (27.0)	χ²=1.17	0.279

### 3.6 Subgroup analyses of overall survival

The subgroup analyses showed a generally consistent direction of association in favor of HDC+HSCT over HDC across most strata. Statistically significant associations were observed among patients with INSS stage IV disease (adjusted HR 0.56, 95% CI 0.36–0.89; P = 0.015), age ≥18 months (adjusted HR 0.58, 95% CI 0.37–0.92; P = 0.020), MYCN non-amplified tumors (adjusted HR 0.56, 95% CI 0.35–0.89; P = 0.016), absence of bone marrow metastasis (adjusted HR 0.55, 95% CI 0.32–0.96; P = 0.035), and adrenal primary tumors (adjusted HR 0.57, 95% CI 0.34–0.96; P = 0.036). No statistically significant treatment-by-subgroup interaction was observed (all P for interaction >0.05) (**Table 6**).

**Table 6:** Subgroup analyses for OS (Cox models; HDC+HSCT vs HDC).

Subgroup	HDC n	HDC+HSCT n	Adjusted HR for OS (95% CI)	P value	P for interaction
INSS stage III	28	30	0.85 (0.40–1.79)	0.668	0.372
INSS stage IV	80	73	0.56 (0.36–0.89)	0.015	0.372
Age <18 months	18	24	0.65 (0.30–1.40)	0.269	0.892
Age ≥18 months	90	79	0.58 (0.37–0.92)	0.020	0.892
MYCN amplified	32	29	0.79 (0.39–1.60)	0.507	0.386
MYCN non-amplified	76	74	0.56 (0.35–0.89)	0.016	0.386
Bone marrow metastasis present	57	51	0.67 (0.38–1.17)	0.159	0.621
Bone marrow metastasis absent	51	52	0.55 (0.32–0.96)	0.035	0.621
Adrenal primary	65	60	0.57 (0.34–0.96)	0.036	0.652
Non-adrenal primary	43	43	0.70 (0.38–1.26)	0.232	0.652

OS, overall survival; HDC, high-dose chemotherapy; HSCT, hematopoietic stem cell transplantation; HR, hazard ratio; INSS, International Neuroblastoma Staging System.

### 3.7 Missing data and sensitivity analyses

Complete-case analyses included 196 of 211 patients (92.9%), with 15 patients (7.1%) excluded from multivariable models due to missingness in at least one prespecified key covariate. Missing data were most frequently observed for tumor biological variables (MYCN amplification status, 1p/11q aberrations, and DNA ploidy) and baseline serum biomarkers (lactate dehydrogenase, neuron-specific enolase, and ferritin), whereas treatment exposure classification (HDC vs HDC+HSCT) and survival outcomes were available for all included patients. For transparency, the proportion of missingness for each covariate is reported in **Supplementary Table 1**. To assess the robustness of the primary findings, multiple imputation by chained equations was performed for key covariates (m=20 imputations), incorporating treatment group, baseline demographic and tumor characteristics, and outcome indicators into the imputation model. In the imputed datasets, the direction and magnitude of the estimated treatment associations were broadly unchanged. Specifically, the survival analyses remained consistent with the complete-case analysis, with higher OS estimates in the HDC+HSCT group and no statistically significant between-group difference in EFS. Similarly, the higher ORR observed in the HDC+HSCT group was preserved, and the comparative patterns for prespecified grade ≥3 toxicities, including bacterial infection, sepsis, mucositis, and renal toxicity, were also broadly unchanged. Overall, these sensitivity analyses supported the internal consistency of the main findings and suggested that incomplete covariate data were unlikely to have materially affected the overall interpretation (**Supplementary Table 2**). No material violation of the proportional hazards assumption was detected for the Cox models of OS or EFS.

### 3.8 Conditioning-regimen heterogeneity and regimen-stratified sensitivity analyses

Within the HDC+HSCT group, conditioning-regimen composition, transplant characteristics, stem cell source, and key supportive care measures are summarized in **Supplementary Table 3**. BuMel and CEM were both used as conditioning regimens, while most patients underwent single autologous transplantation with peripheral blood stem cell support. Supportive care protocols were standardized at the institutional level, with regimen-specific measures applied for busulfan-based conditioning. In regimen-stratified sensitivity analyses, the direction of association for OS favored HDC+HSCT in both the CEM and BuMel strata, although statistical significance was observed only in the BuMel subgroup. EFS remained broadly similar between treatment groups in both strata, and the direction of association for ORR also favored HDC+HSCT (**Supplementary Table 4**). In the full cohort, treatment-by-regimen interaction analyses showed no statistically significant interaction for OS, EFS, or ORR (all P for interaction >0.05; **Supplementary Table 5**), providing no statistically significant evidence that conditioning-regimen heterogeneity materially modified the association between transplantation and outcomes.

### 3.9 Contemporary post-consolidation therapies and additional-adjustment sensitivity analyses

The proportions of patients receiving post-consolidation radiotherapy, anti-GD2 immunotherapy, and retinoid-based maintenance therapy are summarized in **Supplementary Table 6**. Post-consolidation radiotherapy and retinoid-based maintenance therapy were administered in selected patients in both groups, whereas anti-GD2 immunotherapy was not administered in this cohort. To assess whether limited consideration of these variables materially affected the primary survival findings, an additional-adjustment sensitivity analysis was performed by further adding post-consolidation radiotherapy and retinoid therapy to the adjusted Cox models; anti-GD2 immunotherapy was not estimable because there was no variation in exposure. After this additional adjustment, the estimated association between HDC+HSCT and OS remained directionally unchanged and of similar magnitude, and the null association for EFS was likewise preserved (**Supplementary Tables 6–S7**). These findings suggest that the primary survival results were unlikely to be materially explained by imbalance in post-consolidation radiotherapy or maintenance retinoid therapy.

## 4 Discussion

In this retrospective cohort of pediatric patients with advanced-stage neuroblastoma treated with consolidation-intent high-dose therapy, HDC+HSCT was associated with a higher ORR at the first post-consolidation assessment and higher OS estimates than HDC. In contrast, EFS did not differ between groups. Safety analyses showed that severe hematologic toxicity was common in both cohorts, whereas bacterial infection, sepsis, grade ≥3 mucositis, and grade ≥3 renal toxicity were observed more frequently in the HDC+HSCT group. Taken together, these findings suggest that transplant-based consolidation was associated with deeper early response and more favorable survival estimates in this real-world cohort, while also highlighting the importance of structured toxicity mitigation and vigilant supportive care.

The higher ORR observed in the HDC+HSCT group, together with a pattern toward more CR and fewer progressions at the first formal post-consolidation evaluation, is consistent with the clinical rationale for myeloablative intensification followed by stem cell rescue. Contemporary clinical practice frameworks for high-risk neuroblastoma incorporate multimodality therapy across induction, local control, consolidation, and post-consolidation phases, with myeloablative therapy and autologous stem cell rescue remaining a commonly used consolidation strategy in many treatment algorithms. Current guideline-based discussions regard consolidation as a critical treatment phase in which residual chemosensitive disease may be further reduced before subsequent post-consolidation therapy is considered (12, 18, 20, 21). The present cohort-level findings are broadly consistent with this framework, as the transplant group showed a higher ORR after completion of consolidation, although the overall four-category response distribution did not reach conventional statistical significance (22, 23). This apparent discrepancy is not unexpected in a modestly sized cohort, because differences in clinically salient binary endpoints such as ORR (CR+PR) may be detected even when statistical power is insufficient to demonstrate shifts across multiple ordinal response categories simultaneously, particularly when counts in less frequent categories are small (24, 25).

The divergence between OS and EFS is a central point in the interpretation of the present findings. In this cohort, OS estimates were higher in the HDC+HSCT group, whereas EFS, defined to include relapse, progression, second malignant neoplasms, and death, did not differ between groups. Several non-mutually exclusive explanations may account for this pattern. First, EFS events were predominantly relapse and progression in both groups, suggesting that early disease-control failure remained common in both consolidation strategies (26, 27). At the same time, OS and EFS do not necessarily move in parallel in retrospective cohorts, particularly when survival after relapse may vary according to subsequent management, supportive care, or patient fitness at the time of recurrence. Because detailed post-relapse treatment was not analyzed in the present study, differences in survival after an event cannot be excluded as a potential contributor to the observed OS–EFS divergence. Second, EFS is generally more sensitive than OS to surveillance intensity, timing of imaging or bone marrow reassessment, and documentation practices. In a retrospective study, even modest variation in event ascertainment or event dating may have a greater influence on EFS than on OS. Third, treatment-related mortality was uncommon in this cohort and ICU admission was rare; therefore, the higher OS estimates observed in the HDC+HSCT group are unlikely to be explained primarily by early non-relapse mortality differences. Rather, the overall pattern may be more consistent with differences in response depth and/or post-event survival, although these interpretations should remain cautious given the observational design and the absence of detailed salvage-treatment data (28).

These findings should also be interpreted in the context of an evolving therapeutic landscape in which consolidation is increasingly considered alongside post-consolidation immunotherapy and differentiation therapy. Anti-GD2 monoclonal antibodies administered after consolidation have been associated with improved outcomes in high-risk neuroblastoma and are widely incorporated into contemporary treatment frameworks (29, 30). However, anti-GD2 immunotherapy was not administered in the present cohort because it was not routinely available at our center during the study period. Accordingly, the present results should be interpreted within the treatment context actually delivered in this cohort, namely transplant-based or non-transplant high-dose consolidation followed by follow-up surveillance, with retinoid-based differentiation therapy and/or radiotherapy administered in selected patients according to institutional practice. Against this background, the present findings suggest that transplant-based consolidation was associated with a higher early response rate and higher OS estimates, even though EFS remained unchanged. At the same time, the magnitude, durability, and generalizability of these associations may differ in settings where anti-GD2 therapy is routinely integrated into post-consolidation treatment. Recent evidence syntheses continue to examine outcomes with and without myeloablative consolidation in relation to subsequent immunotherapy, supporting the importance of evaluating consolidation strategies within contemporary multimodality treatment sequences rather than in isolation (9).

The safety profile observed in the HDC+HSCT group warrants careful consideration because toxicity trade-offs are central to consolidation decision-making. Grade 3–4 neutropenia was frequent in both groups, reflecting the intensity of consolidation therapy; however, the HDC+HSCT group also showed higher rates of documented bacterial infection, sepsis, grade ≥3 mucositis, and grade ≥3 renal toxicity. These findings are broadly consistent with the recognized early toxicities of transplant-based consolidation, which are commonly linked to profound cytopenias, mucosal barrier injury, and cumulative exposure to potentially nephrotoxic conditioning agents, antimicrobials, and supportive treatments. The longer hematologic recovery time observed in the HDC+HSCT group should not be interpreted as being caused by stem cell infusion itself, which is intended to restore hematopoiesis after myeloablative therapy (31). Rather, this finding more likely reflects the overall transplant-based consolidation course, including the aplastic interval before engraftment, interpatient differences in marrow reserve, prior cumulative treatment exposure, infused stem cell dose, and transplantation-related complications (32). Recent literature has continued to describe infection and mucositis as important early complications in transplant-based approaches for high-risk neuroblastoma, including tandem autologous transplantation strategies (33). The numerically higher incidence of VOD/SOS in the HDC+HSCT group, although not statistically significant in the present study, also remains clinically relevant because SOS/VOD is a well-recognized complication after myeloablative conditioning (34). In addition, conditioning-regimen selection may influence toxicity patterns, and contemporary evidence syntheses comparing busulfan/melphalan with carboplatin/etoposide/melphalan have highlighted renal toxicity and SOS/VOD among clinically meaningful safety outcomes (35). Taken together, the present cohort data suggest that any potential survival advantage associated with transplant-based consolidation should be interpreted alongside its greater acute toxicity burden. These findings further support the importance of standardized antimicrobial prophylaxis, close sepsis surveillance, mucositis prevention and supportive management, and renal-protective monitoring in children undergoing transplant-intensified consolidation (36).

Subgroup analyses in this study showed a generally consistent direction of association across the evaluated strata, and no statistically significant treatment-by-subgroup interaction was detected. Although these subgroup findings should be interpreted cautiously because the analyses were exploratory and multiple subgroup comparisons were performed, the absence of significant interaction suggests that the observed association with OS was not confined to a single narrowly defined subset based on stage, age category, MYCN status, bone marrow involvement, or primary tumor site. At the same time, these results should not be interpreted as evidence that treatment effects were truly uniform across all clinical contexts. In routine practice, selection of consolidation strategy may still reflect clinical factors that are not fully captured in retrospective datasets, including response kinetics during induction, comorbidity burden, organ reserve, physician judgment, family preference, and logistical feasibility of transplantation. Therefore, even in the absence of statistically significant interaction, consolidation decisions should remain individualized, and the subgroup findings in the present study are best viewed as supportive rather than definitive.

Several limitations should be acknowledged. First, owing to its retrospective observational design, selection bias, confounding by indication, and residual confounding cannot be fully excluded despite multivariable adjustment and sensitivity analyses. Group assignment was based on the consolidation strategy actually delivered in routine practice, and unmeasured factors such as early treatment response, minimal residual disease status, transplant eligibility assessment, and clinician judgment may have influenced both treatment selection and outcomes. Second, treatment heterogeneity may have affected the observed associations, including variation in conditioning regimens, supportive care measures, and selected post-consolidation therapies. Although key clinicopathological variables were collected, some potentially important prognostic factors may not have been fully captured in the available records, which could have limited risk adjustment. Third, event-free survival ascertainment may have been influenced by differences in surveillance intensity and documentation timing, even within a single center. Fourth, post-relapse therapies were not comprehensively decomposed, which is relevant because subsequent salvage treatment may influence overall survival in contemporary neuroblastoma management. In addition, toxicity assessment relied on retrospective abstraction of the highest documented Common Terminology Criteria for Adverse Events grade during consolidation, and under-ascertainment of some events remains possible. Finally, the single-center design may limit the generalizability of the findings to other institutions and treatment settings. Despite these limitations, the study provides clinically relevant real-world evidence on the association of consolidation strategies with response, survival, and toxicity in children with advanced-stage neuroblastoma. Future research should prioritize prospective, preferably multicenter, studies or high-quality registry-based analyses with more standardized treatment pathways, comprehensive prognostic assessment, centralized outcome adjudication, and granular characterization of post-relapse therapies, in order to further validate these findings and better define the role of HDC+HSCT within an integrated treatment sequence.

## 5 Conclusion

In this retrospective cohort of children with advanced-stage neuroblastoma, consolidation with HDC+HSCT was associated with a higher ORR and more favorable overall survival estimates than HDC alone, whereas EFS was similar between groups. HDC+HSCT might also be associated with higher rates of selected grade ≥3 toxicities, including bacterial infection, sepsis, mucositis, and renal toxicity. Subgroup and sensitivity analyses generally supported the robustness of these findings, although prospective studies are needed to further validate these observations.

## Data Availability

The raw data supporting the conclusions of this article will be made available by the authors, without undue reservation.
