# Supplementary material for: Efficacy of high-dose chemotherapy combined with hematopoietic stem cell transplantation in advanced neuroblastoma
Source: Front Oncol. 2026 May 14;16:1819260. doi: 10.3389/fonc.2026.1819260 (PMC13215894; doi:10.3389/fonc.2026.1819260)
Supplement: Supplementary file 1 [file Table1.docx]

Supplementary Table S1. Missing data summary and complete-case sample size

| Variable (key covariates for adjusted models) | Missing, n (%) |
| --- | --- |
| Age at diagnosis | 0 (0.0) |
| Sex | 0 (0.0) |
| Body weight | 2 (0.9) |
| Body surface area | 2 (0.9) |
| INSS stage | 0 (0.0) |
| Primary tumor site | 0 (0.0) |
| Metastatic sites (bone) | 0 (0.0) |
| Metastatic sites (bone marrow) | 0 (0.0) |
| Metastatic sites (liver) | 0 (0.0) |
| Metastatic sites (lymph nodes) | 0 (0.0) |
| MYCN amplification status | 6 (2.8) |
| 1p aberration | 7 (3.3) |
| 11q aberration | 8 (3.8) |
| DNA ploidy | 5 (2.4) |
| Baseline lactate dehydrogenase | 4 (1.9) |
| Baseline neuron-specific enolase | 5 (2.4) |
| Baseline ferritin | 6 (2.8) |

Complete-case sample used for adjusted models: n=196/211 (92.9%)

Patients with ≥1 missing key covariate: 15/211 (7.1%)

Abbreviations: INSS, International Neuroblastoma Staging System.

Supplementary Table S2. Sensitivity analysis using multiple imputation (m=20) versus complete-case results

| Outcome | Complete-case analysis (n=196) | Multiple imputation analysis (n=211; m=20) | Consistency (direction/significance) |
| --- | --- | --- | --- |
| OS (HDC+HSCT vs HDC), HR (95% CI), P | 0.62 (0.41–0.94), P=0.024 | 0.60 (0.41–0.90), P=0.014 | Same direction; significant in both |
| EFS (HDC+HSCT vs HDC), HR (95% CI), P | 0.98 (0.70–1.36), P=0.901 | 1.01 (0.74–1.38), P=0.958 | Same direction; non-significant in both |
| ORR (CR+PR), OR (95% CI), P | 1.95 (1.10–3.48), P=0.022 | 2.01 (1.15–3.53), P=0.015 | Same direction; significant in both |
| Grade ≥3 bacterial infection, OR (95% CI), P | 1.86 (1.01–3.43), P=0.046 | 1.92 (1.06–3.46), P=0.032 | Same direction; significant in both |
| Sepsis, OR (95% CI), P | 6.85 (1.43–32.9), P=0.016 | 7.34 (1.65–32.6), P=0.009 | Same direction; significant in both |
| Grade ≥3 mucositis, OR (95% CI), P | 2.04 (1.06–3.93), P=0.032 | 2.12 (1.13–3.96), P=0.024 | Same direction; significant in both |
| Grade ≥3 renal toxicity, OR (95% CI), P | 5.74 (1.16–28.4), P=0.032 | 6.10 (1.38–27.0), P=0.017 | Same direction; significant in both |

Models: OS/EFS estimated by Cox proportional hazards models; ORR and toxicities estimated by logistic regression.

Effect measure: HR for OS/EFS; OR for ORR and binary grade ≥3 toxicities. Reference group = HDC.

Abbreviations: OS, overall survival; EFS, event-free survival; ORR, objective response rate; HR, hazard ratio; OR, odds ratio; CI, confidence interval; HDC, high-dose chemotherapy; HSCT, hematopoietic stem cell transplantation.

Supplementary Table S3. Breakdown of conditioning regimens, transplant characteristics, and key supportive care measures within the HDC+HSCT group

| Variable | HDC+HSCT (n=103) |
| --- | --- |
| Conditioning regimen: BuMel, n (%) | 63 (61.2) |
| Conditioning regimen: CEM, n (%) | 40 (38.8) |
| Single autologous HSCT, n (%) | 96 (93.2) |
| Tandem autologous HSCT, n (%) | 7 (6.8) |
| Peripheral blood stem cell source, n (%) | 98 (95.1) |
| Bone marrow stem cell source, n (%) | 5 (4.9) |
| PK-guided busulfan dosing among BuMel patients, n/N (%) | 58/63 (92.1) |
| Anticonvulsant prophylaxis among BuMel patients, n/N (%) | 63/63 (100.0) |
| Antibacterial prophylaxis, n (%) | 101 (98.1) |
| Antifungal prophylaxis, n (%) | 96 (93.2) |
| G-CSF support, n (%) | 103 (100.0) |
| Transfusion support protocol applied, n (%) | 103 (100.0) |
| VOD/SOS prophylaxis among BuMel patients, n/N (%) | 60/63 (95.2) |

HSCT, hematopoietic stem cell transplantation; BuMel, busulfan/melphalan; CEM, carboplatin/etoposide/melphalan; G-CSF, granulocyte colony-stimulating factor; VOD/SOS, veno-occlusive disease/sinusoidal obstruction syndrome.

Supplementary Table S4. Regimen-stratified sensitivity analyses for survival and response

| Regimen stratum | HDC n | HDC+HSCT n | Adjusted HR for OS (95% CI) | P value | Adjusted HR for EFS (95% CI) | P value | Adjusted OR for ORR (95% CI) | P value |
| --- | --- | --- | --- | --- | --- | --- | --- | --- |
| CEM | 61 | 40 | 0.79 (0.47–1.34) | 0.385 | 1.06 (0.69–1.63) | 0.793 | 1.60 (0.80–3.19) | 0.184 |
| BuMel | 47 | 63 | 0.49 (0.29–0.84) | 0.009 | 0.89 (0.57–1.39) | 0.607 | 2.28 (1.06–4.89) | 0.034 |

Models were adjusted for age at diagnosis, sex, INSS stage, MYCN status, bone marrow metastasis, and primary tumor site. Reference group = HDC within the same regimen stratum.

OS, overall survival; EFS, event-free survival; ORR, objective response rate; HR, hazard ratio; OR, odds ratio; CI, confidence interval.

Supplementary Table S5. Treatment-by-regimen interaction analysis in the full cohort

| Outcome | Main effect of HDC+HSCT vs HDC, adjusted effect estimate (95% CI) | P value | P for HSCT × regimen interaction | Interpretation |
| --- | --- | --- | --- | --- |
| OS | HR 0.62 (0.41–0.94) | 0.024 | 0.254 | No significant interaction |
| EFS | HR 0.98 (0.70–1.36) | 0.901 | 0.561 | No significant interaction |
| ORR | OR 1.95 (1.10–3.48) | 0.022 | 0.472 | No significant interaction |

Interaction models were adjusted for age at diagnosis, sex, INSS stage, MYCN status, bone marrow metastasis, and primary tumor site. Regimen was coded as BuMel vs CEM.

Supplementary Table S6. Post-consolidation radiotherapy, immunotherapy, and retinoid-based maintenance therapy by treatment group

| Variable | HDC (n=108) | HDC+HSCT (n=103) | P value |
| --- | --- | --- | --- |
| Radiotherapy after consolidation, n (%) | 48 (44.4) | 52 (50.5) | 0.377 |
| Anti-GD2 immunotherapy after consolidation, n (%) | 0 (0.0) | 0 (0.0) | NE |
| Retinoid-based maintenance therapy after consolidation, n (%) | 34 (31.5) | 37 (35.9) | 0.494 |

HDC, high-dose chemotherapy; HSCT, hematopoietic stem cell transplantation; NE, not estimable.

Supplementary Table S7. Additional-adjustment sensitivity analysis for survival after further adjustment for post-consolidation therapies

| Outcome | Primary adjusted model HR (95% CI), P | Additional-adjusted model HR (95% CI), P | Interpretation |
| --- | --- | --- | --- |
| OS (HDC+HSCT vs HDC) | 0.62 (0.41–0.94), P=0.024 | 0.64 (0.42–0.97), P=0.034 | Same direction; materially unchanged |
| EFS (HDC+HSCT vs HDC) | 0.98 (0.70–1.36), P=0.901 | 1.00 (0.72–1.40), P=0.981 | Same direction; materially unchanged |

OS, overall survival; EFS, event-free survival; HR, hazard ratio; CI, confidence interval; HDC, high-dose chemotherapy; HSCT, hematopoietic stem cell transplantation.
